# Initial combination versus early sequential standard therapies for Infantile Epileptic Spasms Syndrome—Feedback from stakeholders

**DOI:** 10.1002/epi4.12895

**Published:** 2024-01-13

**Authors:** Praveen Kumar Ramani, Christina Briscoe Abath, Stephanie Donatelli, Aristides Hadjinicolaou, Sebastian Vega Toro, Keryma Acevedo, Karina Rosso Astorga, Kaajal Parbhoo, Avantika Singh, Eva Catenaccio, Puneet Jain, Jitendra Kumar Sahu, Debopam Samanta, Chellamani Harini

**Affiliations:** ^1^ Department of Pediatrics, Division of Pediatric Neurology University of Arkansas for Medical Sciences Little Rock Arkansas USA; ^2^ Division of Pediatric Neurology Children's Hospital of Philadelphia, Perelman School of Medicine at the University of Pennsylvania Philadelphia Pennsylvania USA; ^3^ Department of Neurology, Boston Children's Hospital Harvard Medical School Boston Massachusetts USA; ^4^ Division of Neurology, Department of Pediatrics, Comprehensive Epilepsy Program CHU Sainte‐Justine Montreal Quebec Canada; ^5^ Pediatric Neurology Unit, Department of Pediatrics Hospital Carlos Van Buren Valparaíso Chile; ^6^ Pediatric Neurology Unit, Division of Pediatrics Pontificia Universidad Católica de Chile Santiago Chile; ^7^ Division of Paediatric Neurology Nelson Mandela Children's Hospital Johannesburg South Africa; ^8^ Department of Neurology, Division of Child Neurology Medical College of Wisconsin Milwaukee Wisconsin USA; ^9^ Division of Neurology, Department of Pediatrics, Epilepsy Program Hospital for Sick Children Toronto Ontario Canada; ^10^ Pediatric Neurology Unit Postgraduate Institute of Medical Education & Research Chandigarh India

## 1 SUMMARY

We read with great interest the report by Sourbron et al.^1^ entitled “Medical Treatment in Infants and Young Children with Epilepsy: Off‐label Use of Antiseizure Medications. Survey Report of ILAE Task Force Medical Therapies in Children”. We want to congratulate the authors on this timely article that explores the occurrence of off‐label anti‐seizure medicine use. It analyzes the prescription behavior of over 500 neurologists worldwide in six different epilepsy syndromes, including Infantile Epileptic Spasms Syndrome (IESS).

In this letter, we aim to focus on recommendations concerning the management of infants with IESS. Specifically, we want to address the authors' recommendation that a standard of care should involve combination therapy (hormonal therapy combined with vigabatrin) at the onset of treatment. This recommendation primarily relies on the short‐term clinical remission of epileptic spasms (ES) observed between day 14 and day 42 of treatment, as noted in the prospective randomized controlled International Collaborative Infantile Spasms Study (ICISS). This trial compared combination therapy versus hormonal therapy alone for new‐onset IESS.^2^

We argue that, for IESS, early sequential therapy ‐initiating treatment with standard monotherapy (prioritizing hormonal therapy) and adding another standard therapy (such as vigabatrin) if no electroclinical remission by 14 days‐ is a reasonable alternative to combination therapy. This approach is supported by the available evidence and may be preferable in specific contexts.

## 2 BODY OF REVIEW

We employ the four‐quadrant approach of the medical ethics model, which includes medical indications, patient preferences, quality of life, and contextual factors, to assess this recommendation.^3^

### 2.1 Medical indications: First quadrant

First, we aim to discuss the medical indications for initial combination therapy based on the “Four Quadrant approach,” considering both short‐ and long‐term improvements. Regarding short‐term improvements, a specific univariate stratified analysis from the ICISS study revealed that infants categorized as lower risk for developmental impairment significantly benefited from combination therapy, with ES ceasing in 62% of infants on hormonal therapy alone compared to 88% of infants on combination therapy (*p* < 0.001). However, within the group initially presumed to be at higher risk for developmental impairment at randomization (a proxy for etiology), the cessation of ES occurred in 52% of infants on hormonal therapy versus 58% of infants on combination therapy (*p* = 0.36). Therefore, the effects of combination therapy on ES cessation were not significantly improved in infants considered to be at higher risk for developmental impairment. Based on these results, in the first scenario of IESS (described in the article by Sourbron et al.) where etiology of IESS was related to prenatal injury, combination therapy may not be superior to hormonal therapy alone in achieving short‐term electroclinical remission.

More importantly, the primary rationale for implementing combination therapy extends beyond achieving short‐term clinical remission of ES. Rather, clinicians seek to improve long‐term developmental and epilepsy outcomes. Long‐term follow‐up data from ICISS showed that initial differences in remission do not translate to better long‐term (18‐month) developmental or epilepsy outcomes.^4^ Mean developmental scores calculated using Vineland Adaptive Behavioral scales (VABS) did not differ significantly between the combination therapy group and the hormonal therapy alone group (73.9 vs. 72.7, *p* = 0.55). Even among children considered to be at a lower risk for developmental impairment (where the combination therapy had a superior short‐term outcome), the mean developmental scores in the combination therapy group were not significantly different from those in the hormonal therapy alone group (*p* = 0.15). Additionally, regarding the 18‐month epilepsy outcome, approximately 30% of patients experienced active epilepsy with persistent seizures, and 15% continued to endure ES, with no notable disparity between the groups.

It would be reasonable to surmise that the comparable long‐term results seen in the ICISS study could be attributed to early sequential therapy. In the ICISS trial, 74% of the nonresponders (61 out of 83 infants) to hormonal therapy received vigabatrin by the end of the third month of the trial. Other studies assessing sequential therapy utilizing outcome measures like the ICISS study (cessation of clinical spasms starting at day 14 and sustained for at least 28 days) have demonstrated a similar treatment response to combination therapy. For instance, a prospective study using sequential therapy with vigabatrin followed by prednisolone (if required) yielded an electroclinical remission rate of 72.7%.^5^ Additionally, a retrospective study by Al‐Shehhi et al.^6^ reported an electroclinical response rate of 77% after sequential therapy. Another quality improvement study of sequential therapy (prioritizing initial hormonal therapy over vigabatrin followed by routine initiation of a second treatment after 14 days from the failed first treatment) ultimately resulted in a remission rate of 75.9% within 3 months.^7^

In the future, early sequential therapy may become another reasonable treatment option for combination therapy for clinicians. A recent study evaluating the treatment response to initial standard therapy (ACTH, prednisolone, or vigabatrin) showed that remission can be identified as early as treatment day 7 in nearly 80% of responders. This finding potentially could allow for the earlier initiation of sequential treatment by day 7 in the nonresponders.^8^

### 2.2 Quality of life: Second quadrant

Roughly half of IESS patients will have electroclinical remission with hormonal therapy alone. For example, 108 (57%) of 191 patients on hormonal therapy alone had remission in the ICISS trial. Therefore, combination therapy would expose many IESS patients to another medication without added benefit, even in the short term. Furthermore, infants are typically committed to 6 months of vigabatrin therapy upon initiation. All medications have potential side effects, this raises concerns about the quality of life.

There is a higher occurrence of side effects with combination therapy. Although there was no significant difference between the groups in terms of serious adverse reactions, the ICISS trial demonstrated that about 24% experienced sedation in the combined treatment group compared to 2% in the corticosteroid group (*p* < 0.001).^9^ As a result of adverse reactions, a lower dose was given to 17 infants (three in the hormonal therapy group, and 14 in the combination). This sedation effect could be particularly significant for vulnerable children with severe neurological impairments placing them at risk for aspiration.

Although most infants treated with vigabatrin did not develop a movement disorder, the ICISS trial reported movement disorders in 1% and 8% (*p* = 0.002) of those randomized to hormonal therapy versus combination therapy respectively.^9^ In addition, while rare, several case reports have documented potentially life‐threatening symptoms of dysautonomia, movement disorders, and acute encephalopathy with the onset of combination therapy, suggesting a possible potentiation of vigabatrin toxicity when used in combination with hormonal therapy.^10^ It is plausible that movement disorder and other symptoms seen in these cases are not related to combination therapy but rather represent ascertainment bias. However, in most of these cases, the time course of symptom development (typically within a couple of weeks of the onset of combination therapy) and the symptomatic improvement upon discontinuation or reduction of vigabatrin dose or cessation of hormonal therapy suggest a plausible link between combination therapy and an increased risk of vigabatrin toxicity.^11^

Thus, regarding quality of life, sequential therapy is a reasonable alternative to combination therapy.

### 2.3 Contextual factors: Third quadrant

As a group of child neurologists and epileptologists from various countries, including both high‐ and middle‐income countries, we want to highlight further the challenges of combination therapy that can arise based on different healthcare contexts. These encompass societal factors related to resource allocation, healthcare policies, and financial considerations. For instance, the prescription of Vigabatrin is regulated in certain countries like the USA, where it is accessible to healthcare providers and patients only through a specialized program known as the Vigabatrin Risk Evaluation and Mitigation Strategy (REMS) Program. Moreover, in the USA, it is recommended to conduct vision testing before or within 4 weeks after initiating vigabatrin, and at least every 3 months during treatment until discontinuation. These regulations impose an additional burden on families.

In the USA, copayment costs for medications can be significant and variable. The price of vigabatrin can be over $50 000 US for a 6‐month course.^12^ In Chile, most caregivers who use the public system must pay out of pocket for the medications, and costs of dual therapy could be prohibitive and place an economic burden on families. This raises a critical concern regarding the cost‐effectiveness of combination therapy, an aspect that has been inadequately explored thus far and merits further attention.

Implementing combination therapy as a standard nonetheless sets a precedent for the rest of the world. In many countries, including South Africa, Canada, and Chile, there can be an irregular supply of first‐line medications. Implementation of combination therapy would further exacerbate challenges in access in these countries, allocating two medications for some children with IESS and none for others. This could contribute to perceived inequity in treatment by families of children with IESS who reside in regions unable to access medications.

### 2.4 Patient preferences: Fourth quadrant

Based on our literature review, little is known about caregiver preferences with respect to IESS treatment. Further qualitative studies need to be done to understand the parents' perspectives. To promote a shared decision‐making process, it is necessary to incorporate parents and patient representatives in treatment decisions. Given the pressure of time and the need for early cessation of ES, parents could be motivated to passively accept the therapeutic option proposed to them without further questioning. In this sense, it is relevant to share available information and support to the family during the discussion of risks and benefits of combination vs sequential therapy.

## 3 CONCLUSIONS

We used the four‐quadrant model to analyze clinician decision‐making for implementation of sequential versus combination therapy for IESS. We found that from a medical perspective, that combination therapy did not result in better long‐term developmental and epilepsy outcomes. Sequential therapy (prioritizing initial hormonal therapy), with the early addition of a second standard drug (by day 7 or 14) may provide efficient therapy escalation when needed, while minimizing potential adverse effects in early responders.^13^ From a quality‐of‐life perspective and contextual perspective, there also are concerns about increased risk for adverse effects, supply issues, and costs. Further research is needed to better understand caregiver perspectives.

It is possible that combination therapy may benefit a subset of infants with IESS without medical comorbidities or those with long lead times. Further prospective studies can help inform patient subgroups who may benefit most from different treatment approaches. Until that time, sequential therapy with the early addition of a second drug remains an appropriate alternative to combination therapy for treatment of IESS.

## CONFLICT OF INTEREST STATEMENT

The author has no conflicts of interest to disclose. The author has read the Journal's position on issues involved in ethical publication and affirms that this report is consistent with those guidelines.
